# Microscopy of the Dental Tissues. New Series

**Published:** 1868-11

**Authors:** S. P. Cutler


### ARTICLE II.

### *Microscopy of the Dental Tissues. New Series.*

By S. P. Cutler M. D., A. E. G., D. D. S.,

*Continued.*

Histology—It is a well established fact, that oxygen is carried from the lungs to the tissues, and the object of the introduction of the gas is also understood to be a necessary destroying agent that life may be re-enacted repeatedly through the death and destruction of the living organisms. That is to say, that the very destroying agent is the direct agent in restoring and sustaining life, when proper conditions are supplied.

Reduction, on the one hand, of living tissues to their binary inorganic elements; on the other, by the same process (which may be regarded as a double one,) the tissues, when proper elements are supplied, are rebuilt out of the new materials, as old materials are removed by oxygen. Any cause that excludes oxygen from the tissues effectually prevents or arrests both waste and nutrition, even if fully supplied with nutritive materials.

Life manifestations rapidly sink during the time of the exclusion of oxygen, and if too long withheld asphyxia follows.

Any vapor or gas, that is capable of replacing oxygen in the blood by inhalation, and from thence entering the tissues, even if such vapors are chemically combined with oxygen so that no oxidation of the tissues takes place, causes life manifestations rapidly to sink. Anæsthesia produced by vapors may be inferred to act in this manner chiefly when not actually poisonous, (but entirely inert in themselves,) by arresting inter-molecular action which is the direct result of oxidation in the cells.

The nerves and brain, being only the factors of forces generated in other tissues, are also reservoirs, depositories and distributors of such forces when supplied by molecular action of all the tissues, the nerves themselves included. The brain and nerves expend the force, as supplied by the tissues, in the various mental and nerve phenomena.

As the magnetic force of the galvanic battery, is developed by oxidation, so in a similar manner, nerve force is developed by oxidation, and every where in the proximity of nerve terminations, received and transmitted through the sensorial branches to the brain and nerve centres, are there held in reserve for future use, to be transmitted through the motor nerves.

There must be positive and negative poles meetiug in order to complete a magnetic circle, hence the identity of nerve force and magnetic force may be inferred. It is evident then, if the above hypothesis be true, that life forces are developed in the innumerable cells of the tissues, including the blood as a moving and living tissue, carrying in its sanguine currents both live and dead matter, one *into* and the other *out* of the system. Any interrupting cause to cell oxidation must necessarily cause functional disturbance of the tissue or part so disturbed. Oxidation and nutrition may be regarded as balancing forces when in equilibrium. Believing that the cells are made up of extensively perforated walls caused by intermolecular apertures, owing to want of perfect approximation of molecules, I can readily account for the entrance and escape of liquids and gases into and out of the cells. Gases and liqnids that have affinities for the cells, may readily find unobstructed entrance, such as nutrition in a state of solution and free oxygen. These elements must be drawn as well as forced from the circulation into the tissues and cells passing through the capillary walls by innumerable openings.

When these affinities that invite the nutritive elements to the tissues have been compensated by oxidation, then the products of such oxidation are waste matters no longer having any afflnity for the cells, there is a mutual repulsion which facilitates their rejection or transudation to the outer world as debris. To be more explicit. Food is not forced into the system by any outward force pump, neither is free oxygen; on the contrary, they are drawn in by internal forces, which are self regulating, and the waste matters are urged outwardly by a repelling force from within, and not drawn out by any suction pump from without. This double current then is carried on by harmonious laws from within acting in harmony with the outer world. Oxygen as it is taken into the system is inert or inactive, and does not readily combine with the cells, and must become ozone rendered active before any union or combination can take place; the gas itself must begin to condense and give up a portion of its caloric to be rendered sensible, or free caloric, before any union or compound can be formed. The only way I can readily account for this conversion of oxygen, is by its condensation as it passes through the innumerable minute apertures of the cell walls, causing the gas to give up a portion of latent caloric to the cell; the gas then becoming active combines with carbon and hydrogen of the tissues, molecule uniting with molecule, that is, a molecule of oxygen uniting molecule by molecule with both carbon and hydrogen of the cells, the oxygen condensing to form water and carbonic acid, and giving up its latent heat to the tissues so acted upon as to keep up animal temperature, at the same time generating nerve force, (whatever it may be,) resulting from thermal disturbance the same as the magnetic battery. The cells are built chiefly of four elements, carbon, hydrogen, oxygen and nitrogen. We have then oxygen already combined in the cell structure, still that does not prevent farther oxidation by the presence of free oxygen, as it is being converted into nascent ozone by its pairing off into binary inorganic elements, with the carbon and hydrogen of the cells, resulting in the formation of water and carbonic acid.

The oxygen, already forming an element of the tissues, and nitrogen are liberated into nascent oxygen and nitrogen from a demi solid state into gases, which conversion or change requires additional caloric, which must come from the free oxygen that enters and combines with the tissues, causing their continual disintegration on the one hand, and a corresponding nutrition on the other. The condensation of the fluid plasma into demi solid tissue gives up a certain amount of heat of liquidity to become a solid, (supposing this to be a chemical condensation.) The oxygen and nitrogen of the tissues after the carbon and hydrogen are displaced, are necessarily set free and are in a nascent state, and we may suppose that new chemical compounds will be formed, such as ammonia by nitrogen uniting with a portion of liberated and unoxidized hydrogen, the liberated oxygen combining with a certain portion of the liberated unoxidized carbon forming carbonic acid, which may unite with ammonia to form carbonate of ammonia which is readily soluble in the water that may be present. This ammonia may undergo further changes before leaving the system not necessary to mention.

I maintain that when any one element is removed from a cell, just its combining number of the other elements are also liberated, and can no longer remain as part of the cell structure and has to be replaced by new plasma, and not patched up as any piece of machinery may be. Just as soon as the carbon, which is a solid and forms the solid basis of the fleshy tissues, is removed, the other three being gaseous, at once absorb sufficient caloric to reconstruct them into gases again, as already explained.

Disintegration and integration travel on paripassee, and by these perpetual changes going on, thermal disturbances keep up animal heat and nerve or vital force, without which no life manifestations could be enacted. Life forces then may be conceived to originate in cell action, both in the solids and in the blood cells. On the other hand, any cause as above stated, that arrests oxidation in the cells, suspends life functions, just in proportion to that arrestation. Life then is motion, and death is inertia in the cells. Every cell may be regarded as a vital or life point, and as every part of the organisms is made up of cells, except the fluids or all the elements in a fluid condition, their numbers are beyond estimation.

The vital manifestations are carried on within certain limits, and any disturbing cause acting with sufflcient energy from without or within, continued a sufficient length of time, may produce disease, either local or general. On the one hand retarded cell activity may result in chill, on the other hand, exalted cell action may result in fever, these conditions may alternate each other, as in case of chills and fever, notwithstanding, nutrition and oxygen may be carried in, in normal quantities. There are certain vapors and gases that are respirable that may be inhaled with air, that will partially arrest oxidation in certain quantities and in certain other quantities will entirely suspend cell action, producing anæsthesia which if too long continued may cause death. There is sufficient reserved vital force retained in the braiu and nerve centres to sustain life an indefinite time after arrestation of cell action.

